# Oxford Lithium Trial (OxLith) of the early affective, cognitive, neural and biochemical effects of lithium carbonate in bipolar disorder: study protocol for a randomised controlled trial

**DOI:** 10.1186/s13063-016-1230-7

**Published:** 2016-03-02

**Authors:** Kate E. A. Saunders, Andrea Cipriani, Jennifer Rendell, Mary-Jane Attenburrow, Natalie Nelissen, Amy C. Bilderbeck, Sridhar R. Vasudevan, Grant Churchill, Guy M. Goodwin, Anna C. Nobre, Catherine J. Harmer, Paul J. Harrison, John R. Geddes

**Affiliations:** Department of Psychiatry, Oxford University, Oxford Health NHS Foundation Trust , Warneford Hospital, Oxford, OX3 7JX United Kingdom; Department of Pharmacology, Oxford University, Mansfield Road, Oxford, OX1 3QT United Kingdom

**Keywords:** Bipolar disorder, Mood instability, Lithium, Experimental medicine

## Abstract

**Background:**

Despite lithium’s being the most effective drug for bipolar disorder and in clinical use for decades, we still know very little about its early effects relevant to its mode of action.

**Methods/design:**

The Oxford Lithium Trial is a double-blind, randomised, placebo-controlled study of 6-week lithium treatment in participants with bipolar disorder and mood instability. Its aim is to identify early clinical, neurocognitive and biological effects. Participants (*n* = 40) will undergo an intensive battery of multi-modal investigations, including remote monitoring of mood, activity and physiology, as well as cognitive testing, fMRI and magnetoencephalography, together with biochemical and gene expression measurements to assess renal, inflammatory and circadian effects.

**Discussion:**

The findings derived from this trial may be of value in predicting subsequent therapeutic response or side effects, not only relevant to the use of lithium but also providing a potential signature to help in more rapid evaluation of novel mood stabilisers. In this respect, OxLith is a step towards the development of a valid experimental medicine model for bipolar disorder.

**Trial registration:**

ISRCTN91624955. Registered on 22 January 2015.

## Background

Bipolar disorder is a mood disorder which affects around 2 % of the population worldwide [1]. Lithium remains the only pharmacological agent primarily indicated for the treatment of bipolar disorder and has a robust evidence base for efficacy, especially in the long term [2, 3]. It also significantly reduces suicidal behaviour [4]. However, lithium treatment has serious limitations, including toxicity in overdose, many side effects and lack of efficacy in some participants [5]. New treatments are urgently needed because, despite its efficacy, many participants do not respond to or cannot tolerate lithium, or have to stop the drug due to toxicity. Moreover, the depressive episodes of bipolar disorder are difficult to treat [6] and are often prolonged, and, even when not meeting criteria for a depressive or manic episode, participants often have residual mood instability, cognitive difficulties and other complaints [7]. Despite the widespread use of lithium in bipolar disorder for over 50 years, we still know little about its mechanism of action or about the early predictors of therapeutic response. Such information would be of value, both for optimising the clinical utility of lithium and in providing potential early markers of efficacy which could be targeted in investigations of novel candidate mood stabilisers. At present, such investigations require lengthy clinical trials which are expensive and therefore risky [8, 9]. For example, the researchers in the BALANCE trial, comparing lithium, divalproex and combination lithium plus divalproex in the prevention of relapse in bipolar disorder, followed participants for up to 2 years after randomisation, starting in 2000 and reported in 2010 [10]. The field badly needs more proximal, early or intermediate outcomes that can be used reliably in early-phase trials to provide initial evidence of efficacy. Together, the absence of validated targets and the consequent difficulties of conducting clinical development programmes contribute to the dearth of therapeutic innovation in this field.

Further, as more has been discovered about the typical course of bipolar disorder, it has become clear that the definition of bipolar disorder as an episodic condition in which depression and mania are interspersed with normal mood (and its implication of normal functioning) is over-simplistic and potentially misleading [11]. More commonly, people with bipolar disorder experience prominent inter-episodic sub-syndromal mood symptoms [12, 13]. Indeed, there is increasing evidence that chronic mood instability, which persists between clinically significant mood episodes, is an important component of the clinical picture [12]. Self-reported mood instability is a risk factor for the emergence of bipolar disorder [14] and often is a disabling feature for people with bipolar disorder, particularly in those of young age or at the beginning of their illness [15]. Reactivity of mood to external events is also greater in bipolar disorder compared with other mood disorders [16]. We have demonstrated the realities of mood instability in participants, and how it can be captured longitudinally at scale and over long periods of time, using a mood-monitoring system called True Colours (https://oxfordhealth.truecolours.nhs.uk/www/en/), [17]; other groups also have provided similar evidence [18, 19]. The Oxford Lithium Trial (OxLith) will take place within our ongoing programme of research, the Collaborative Network for Bipolar Research to Improve Outcomes (CONBRIO), in which we are exploring the nature and significance of mood instability.

On the basis of these data and considerations, we hypothesise that persistent mood instability is a risk factor for clinically significant mood episodes and that better mood stabilisation may improve outcomes. If true, and if robust markers of mood instability can be identified, this raises the possibility that measuring a change in mood stability in response to lithium over a short period such as a few weeks could predict long-term therapeutic efficacy). In turn, this would be a major contribution to the development of an experimental medicine model allowing more rapid and efficient early-phase testing of novel treatments, helping to overcome the current impasse. A precedent is provided by the Oxford Emotional Test Battery (ETB) [20], which facilitates early detection of (and gives a cognitive model for) anti-depressant efficacy. The ETB is a set of emotion-related paradigms that have been shown to be sensitive to the acute effects of anti-depressants and is now in use in development programs for novel anti-depressant drugs. Importantly, the ETB works in healthy subjects as well as in participants with depression, enhancing its utility such that, if our hypothesis is correct, novel bipolar disorder treatments could also be tested in non-clinical populations selected for high mood instability.

**Fig. 1:**
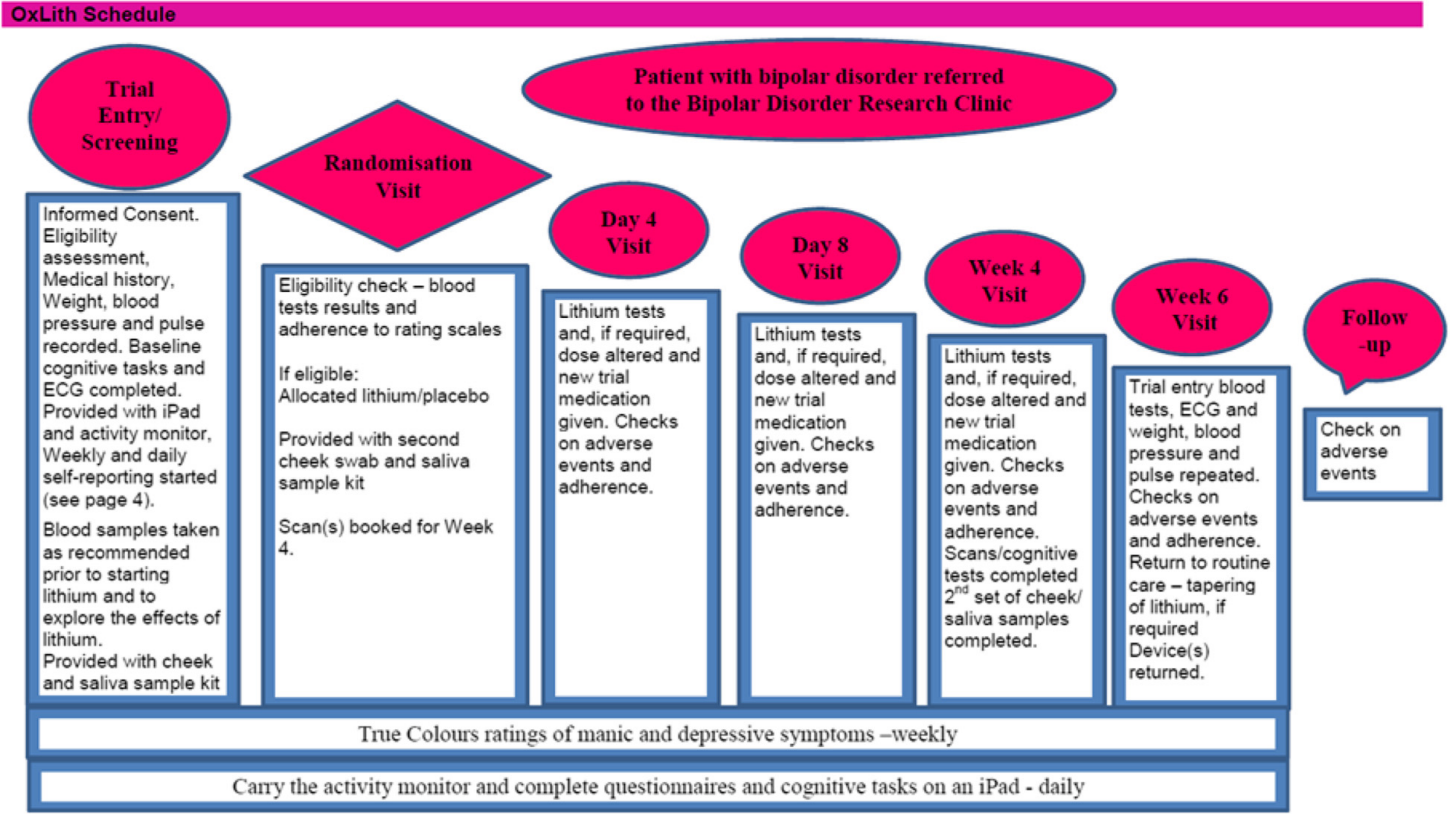
OxLith trial schedule. ECG electrocardiogram

We have designed OxLith, an innovative randomised controlled trial, to investigate the effects of lithium on mood stability and on a wide range of cognitive, neural and physiological indices. OxLith will take place within CONBRIO, a research programme funded by the Wellcome Trust, which is seeking to (1) determine the nature and significance of mood instability, its physiological correlates, and the factors influencing it; (2) find novel ways to measure mood stability in routine clinical practice using a range of measures and devices; (3) apply mathematically sophisticated methods for data analysis and interpretation; and (4) develop an experimental medicine model for mood stabilisation and bipolar disorder.

## Methods/design

The schedule for the OxLith trial is displayed in Fig. 1.

### Study objectives

The study objectives are as follows:

1. To compare the effects of lithium and placebo on mood instability
2. To measure the cognitive effects of lithium and to look for correlations with current mood state
3. To explore the effects of lithium on the variability in neural dynamics during magnetoencephalography (MEG) and functional magnetic resonance imaging (fMRI) scans
4. To explore the effects of lithium on physical activity and sleep
5. To explore the short-term effects of lithium on gene expression; circadian hormones; and thyroid, parathyroid and renal function

### Study design

The trial is designed as a single-centre, prospective, randomised, double-blind, placebo-controlled trial. Eligible participants will be randomised in a 1:1 allocation to lithium or placebo. The randomisation algorithm will minimise separately on two variables age (<25 and ≥25 years) and sex (male, female) which are related to prognosis. Routine psychiatric follow-up with be arranged outside the trial as required. Participants will be randomised following a 2-week run-in period and take the study drug for 6 weeks.

### Ethics

Written consent will be obtained from all participants. The consent form will include agreement for the results of any blood tests performed before consent to be used as baseline measures for the trial, negating the need for additional venepuncture. The study protocol was approved on 14 April 2015 by the National Research Ethics Service Committee South Central – Oxford A (reference number 15/SC/0109) and on 10 July 2015 by Oxford Health NHS Foundation Trust.

### Participants and recruitment

Participants will be recruited from the bipolar disorders research clinic in Oxford. All participants will have a diagnosis of bipolar disorder (I, II or not otherwise specified) based on the *Diagnostic and Statistical Manual of Mental Disorders, Fourth Edition,* criteria and will report clinically significant mood instability. In addition, they will be individuals for whom there is uncertainty about the benefits of lithium (for example, for an individual who has recently been diagnosed or has had relatively few major mood episodes). Participants will be aged 18 years or older; will have pre-treatment renal, cardiac, thyroid and parathyroid function acceptable for the initiation of lithium; and will be willing and able to consent to participate in the trial. Exclusion criteria include any contraindication to taking lithium, concomitant psychotropic medication which cannot be withdrawn, clinically significant substance abuse, the need for treatment of an acute mood episode where placebo would be unethical, pregnancy or being of childbearing age not using effective contraception, and current suicidal ideation.

### Interventions

The interventions are placebo or lithium carbonate 200-mg prolonged release tablets are to be taken orally at night and titrated to target serum level of 0.7 mmol/L. This dosing is consistent with routine practice.

### Measures

#### Primary outcome

The primary outcome will be mood severity and stability over the 6-week randomised period as measured using a quick inventory of depressive symptoms (16-item Quick Inventory of Depressive Symptoms–Self-Rated), the Altman Self-Rating Mania Scale and daily mood measures recorded using the short form of the Positive and Negative Affect Schedule (PANAS).

#### Secondary outcomes

##### Cognition and neural dynamics

Cognitive task performance and neural dynamics will be measured to identify the effects of lithium on physiological variability and to explore the relationship between this variability and the experience of mood instability. The outcome measures will be (1) cognitive task performance for decision making, implicit and explicit reinforcement learning (completed daily using a smart device) in relation to variability in daily measures of mood (PANAS), and (2) the results of two brain scans, one using MEG and the other fMRI. Both will include scans during resting state and whilst performing a decision-making task similar to one of the daily outcomes. Scans will be performed between 3 and 4 weeks post-randomisation when the lithium level has reached a steady state.

##### Sleep and motor activation

Participants will wear an ActiGraph monitor (ActiGraph, Pensacola, FL, USA) for the duration of the study. This will allow us to monitor daytime activity and sleep patterns.

##### Circadian system

We will monitor circadian gene expression (including *BMALL *, *PER 1* and *PER 2*) and genes targeted by lithium (e.g., *GSK3*, *IMP* and *GAD L1*) collected from the cheek. Circadian controlled hormones (cortisol and melatonin) will also be collected from saliva to explore the relationship between the circadian system, mood stability and lithium. Gene expression, melatonin and cortisol levels will all be measured every 4 h over two separate 32-h periods, one prior to randomisation and the other between weeks 3 and 4 post-randomisation.

##### Physiological effects of lithium

Blood samples will be collected pre-randomisation and at the end of the randomised phase to explore changes in a number of known biomarkers related to thyroid (thyroid-stimulating hormone, thyroid antibodies, triiodothyronine and free thyroxine), parathyroid (parathyroid hormone, calcium and vitamin D) and renal function (urea, creatinine, potassium, glomerular filtration rate, neutrophil gelatinase-associated lipocalin and cystatin C). Blood samples will also be used to explore lithium-associated changes in intracellular protein composition and structure.

### Power analysis and sample size

The target sample size is 40 participants (20 of whom will be allocated to treatment with lithium and 20 to placebo). This sample size will give more than 90 % power to detect differences between groups on the main mood outcomes at a significance level of 0.5 %. Pragmatically, this sample size is achievable.

### Blinding

All trial psychiatrists and participants will be blinded. For those participants taking placebo, sham lithium level results will be presented to the trial psychiatrist by an unblinded researcher. The sham results will be based upon information about reported adherence, time since most recent dose, and adverse events. At or just before the 6-week visit, the unblinded researcher will reveal the participant’s allocated treatment to enable the participant and psychiatrist to discuss treatment options. In the event of a medical emergency, routine care for a patient who may have been taking lithium and has symptoms consistent with lithium toxicity would be to obtain a lithium level immediately, thus negating the need for the treating doctor to be informed about the trial allocation.

### Statistical analysis plan

Multiple pre- and post-randomisation mood measurements will be used in an analysis of covariance model to compare lithium with placebo. Methods from the fMRIB Statistical Library (FSL) will be used to analyse fMRI data. The FSL is a software library containing image analysis and statistical tools for functional, structural and diffusion magnetic resonance imaging brain data. MEG data will be analysed using a generalised linear model (GLM) and analysis of variance. The GLM method is a standard non-linear beam former used to determine the time course of neuronal activation for each point in a predefined source space. Actigraphy data will be correlated with mood and cognitive data.

In addition to these more traditional approaches to analysis, we will apply linear and non-linear time series methods and other advanced mathematical approaches, including machine learning. These techniques can produce binary or multi-level classifications as well as point and density forecasts to quantify confidence intervals. We will also apply the mathematical theory of rough paths [21], providing a method to reduce datasets from complex non-linear systems subjected to rapidly fluctuating stimuli to their critical information, facilitating classification and prediction.

### Dissemination

Regardless of the magnitude or direction of effect, the main findings of the trial will be compiled and published in an appropriate journal and made available to participants.

## Discussion

OxLith has two main goals. The first is to better characterise the early effects and mechanism of action of lithium, together with its tolerability profile, compared with placebo. The second is to establish whether mood instability, of a higher frequency than clinical episodes of depression or mania, is sensitive to lithium. The exploratory design of OxLith is intended to provide a paradigm and generate new experimental medicine outcomes and methods which can be used to expedite the development of new medicines for bipolar disorder. Drugs such as ebselen which have effects similar to those of lithium in vitro and in available mouse models of bipolar disorder are likely early candidates for such approaches.

### Trial status

Recruitment has started. The duration of the study is 3 years.

## Abbreviations

CONBRIO: Collaborative Network for Bipolar Research to Improve Outcomes
ECG: E lectrocardiogram
ETB: Oxford Emotional Test Battery
fMRI: F unctional magnetic resonance imaging
FSL: fMRIB Statistical Library
GLM: G eneralised linear model
MEG: M agnetoencephalography
OxLith: Oxford Lithium Trial
PANAS: Positive and Negative Affect Schedule
